# Fifteen quick tips for success with HPC, i.e., responsibly BASHing that Linux cluster

**DOI:** 10.1371/journal.pcbi.1009207

**Published:** 2021-08-05

**Authors:** Jamie J. Alnasir

**Affiliations:** Department of Computing, Imperial College, London, England, United Kingdom; University of Toronto, CANADA

## Introduction

High-performance computing (HPC) clusters are widely used in-house at scientific and academic research institutions [1]. For some users, the transition from running their analyses on a single workstation to running them on a complex, multi-tenanted cluster, usually employing some degree of parallelism, can be challenging, if not bewildering, especially for users whose role is not predominantly computational in nature. On the other hand, there are more experienced users, who can benefit from pointers on how to get the best from their use of HPC.

This 15 quick tips guide is aimed at helping you identify ways to improve your utilisation of HPC, avoiding common pitfalls that can negatively impact other users and will also help ease the load (pun intended) on your HPC sysadmin. It is intended to provide technical advice common to the use of HPC platforms such as Load Sharing Facility (LSF), Slurm, PBS/Torque, SGE, LoadLeveler, and YARN, the scheduler used with Hadoop/Spark platform. Tips on employing parallelism and optimising your jobs by looking for common resource bottlenecks are also given. Guidance is also provided on utilising workflow languages and package managers to ensure your computational workflows are standardised, platform agnostic, and reproducible. While each of these quick tips is written to stand on its own, they are loosely ordered to be progressively more advanced.

### Tip 1: Do the HPC induction: Read the manual

Our first rule starts with a fitting anecdote to a first encounter with HPC in the first year of my PhD. During lunch with colleagues, and after a lull in the conversation, Bob—the IT support lead for our department—exclaimed: “I just received 14,000 emails thanks to a HPC user when I arrived at my desk this morning,” which of course got the conversation going again with some intrigue and amusement. He was referring to the automatic emails sent out by the department’s HPC cluster’s job scheduler, IBM LSF—it was configured to automatically email the job-submitting user an email report on job completion [2]. Failing that, e.g., when the user’s inbox is already full, it is set up to email the report to the designated sysadmin. It transpired that a certain user—yes, you’ve guessed correctly, it was me—submitted a very large number of jobs (1 for each entry in the Protein Data Bank), but had not overridden the default setting to email the job report back to the user.

While the automatic emailing of job status reports as a default is not a common configuration, it’s not unheard of. It wasn’t immediately obvious to me that I needed to specify an output filename using the -o option to *bsub*, in order to suppress the job report email and write the output to a file instead. Hence, the first rule here is the following: 1. Do the HPC induction: Read the manual.

The cluster I had used belonged to a small research group. It had only a few users, and there wasn’t any induction training or manual. However, most institutions provide an HPC induction and training material, often also available via an institutional intranet, because these HPC clusters are typically multi-tenanted systems, and improper use can negatively impact other users. It makes sense to make sure you do this before you commence. The HPC scheduler user manual will usually be found online and should be consulted for more detailed information. Typically, this documentation will contain examples of common job submission and management tasks with their parameters.

### Tip 2: Brush up on your Linux commands and BASH

Bourne Again SHell (BASH) is a shell—a command-line interpreter language—whose name is a pun on Stephen Bourne, the developer of the UNIX shell, sh which BASH descends directly from [3]. A shell provides access to the operating system’s (OS) services. Areas to which particular focus should be given include, for example,

- **Streaming, piping, and redirection**: these facilitate the control of input to and output from processing.
- **Environment variables**: for setting and sharing of parameters between software as well as the OS.
- **Compression and archiving**: usually necessary prior to network transfer, useful for collating large numbers of files in more manageable single archives, and for optimising storage.
- **Regular expressions**: provide powerful search/replace operations on matched character patterns. BASH supports POSIX regular expressions.

A 10 simple rules article that covered getting starting with command-line bioinformatics will be a useful read for those new to the command line, for example, BASH [4]. When familiarising yourself with the HPC scripts—whether BASH or other scripting languages—it can be useful to first run simple “controlled” examples, i.e., by modifying those provided in documentation and then progressively increasing the complexity of the jobs.

### Tip 3: Do not run your commands on a login node

**Fig 1:**
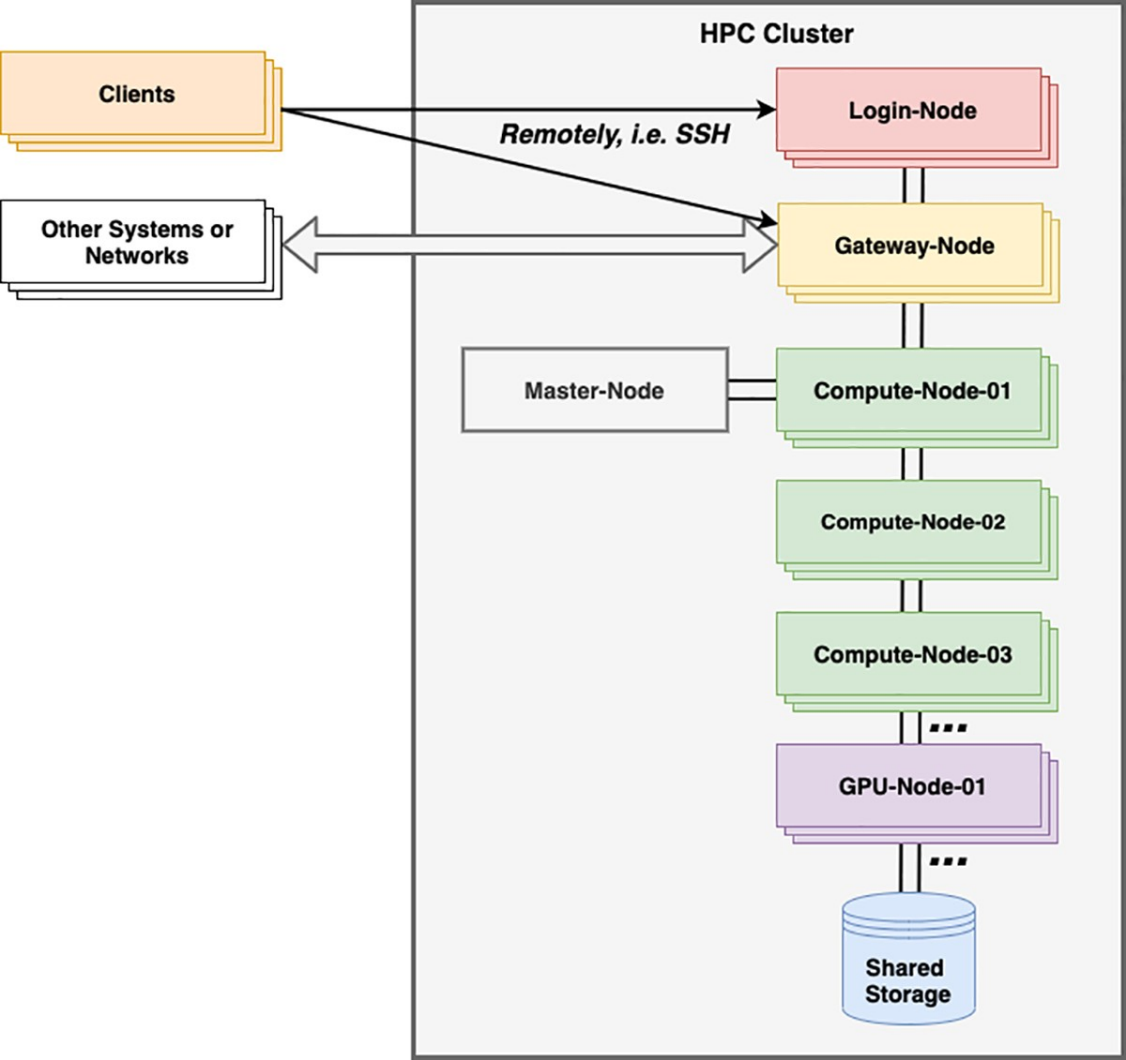
Simplified organisation of a typical HPC system. Users log in remotely via SSH to a login node from a client machine. Jobs are scheduled by job scheduler software running on the master node and run on compute nodes and GPU nodes. Gateway node(s) facilitate the transfer of large files into and out of the HPC system. GPU, graphics processing unit; HPC, high-performance computing; SSH, (Secure Shell Protocol).

There are often one or more login nodes that are reserved to facilitate users logging on to the cluster via SSH (Secure Shell Protocol) and for submitting jobs to the job scheduler, as well as performing non-intensive tasks, such as editing source code and job submission scripts (Fig 1). This is because the load on the cluster is balanced by the job scheduler running on the master node. This is the reason why logging into individual nodes to run jobs directly on them is bad practice and is usually not permitted. Login nodes are typically light-weight virtual machines and are therefore not suitable for running intensive tasks; doing so would slow the login nodes for other users and cause delays in logging in. It is common practice for such tasks to be terminated often without prior notice.

Job schedulers typically provide an interactive job mode where an interactive shell session is initiated on one of the compute nodes and responds to both ad hoc commands and scripts running within the session. This can be used for slightly more intensive tasks such as, e.g., compiling code for HPC, recursively finding files.

### Tip 4: Do not wait for the cluster load to decrease to submit jobs

HPC batch schedulers are designed to simultaneously accept multiple job submissions from multiple users. Typically, these jobs are placed in a queue (or partition in Slurm) to be scheduled for execution. Therefore, although some jobs submitted during increased cluster load will not run straight away, they will be held in the queue until such time as the conditions allow them to be run; hence, there is no need to wait for the load to decrease before submitting your job(s).

### Tip 5: Be aware of the file system

Invariably, HPC systems use distributed file systems across their nodes such as Lustre or General Parallel File System (GPFS), while HDFS is used in Hadoop/Spark clusters. Typically, such file systems are not optimised for large numbers of very small files, which can be found in some routine computational biology tasks (see Quick Tip 13 for a possible solution using a single containerised image for large numbers of small files). As a result, some HPC systems offer temporary storage locally on the nodes for computation—this is often referred to as scratch space. Scratch space is often shared with other users, and so it is important to follow the HPC guidelines and obey any recommended quotas.

### Tip 6: Take care with large file transfers

It will often be necessary to transfer large files into and out of an HPC cluster, e.g., next-generation sequencing data. While HPC nodes are connected with high-speed network interconnects, the available bandwidth is finite and shared by all of the users, their jobs, the OS, and the cluster management software. For this reason, HPC systems often have designated nodes—typically called gateway nodes (see Fig 1)—specifically for file transfers into and out of the HPC cluster, with defined procedures or commands to use.

HPC systems will often have specialist file transfer software to access services such as Globus [5] available and exotic file transfer tools, e.g., Aspera Connect [6] or high-speed command-line tools, e.g., bbcp [7] may also be installed.

Other tips for large file transfers include scheduling them to run, i.e., on the appropriate gateway node or using the correct procedure, during periods of low cluster activity, i.e., at night, or notifying your HPC support team who can perform or schedule them on your behalf.

### Tip 7: Be aware of any recharge model

Some institutions operate a recharge model whereby the computing department that runs the HPC service bills research groups or departments who use the cluster. On an HPC system, it is typically storage and disk space that are charged for. In these cases, central processing unit (CPU) usage is usually charged in CPU/hours for CPU time used—not wall time requested on job submission—and storage charges are usually billed in, e.g., TB/year for exceeding a default user quota. HPC jobs are therefore often submitted with a project code to identify the research group or project, particularly when they are billed for use. So, it is important to know beforehand if a recharge model is in place, especially if you are going to submit a very intensive or long duration job or will exceed your disk quota.

### Tip 8: Do not use Unicode characters in file paths

As much as you may want to use the correct diacritics for your name in your filenames and file paths, try to avoid this if doing so involves the use of uncommon Unicode characters. Likewise, spaces can also be problematic. Disregarding this will create problems for other users trying to access your files and will also create difficulties for the HPC support team when they come to perform nonautomated operations on your files such as, for example, migrations, file transfers, and routine housekeeping.

### Tip 9: Understand the levels of parallelism required by your job

In order to leverage the increased throughput that parallelism inherent in distributed computing offers, it is necessary to understand the levels on which this is applied in executing the computational tasks in your workflow.

**Fig 2:**
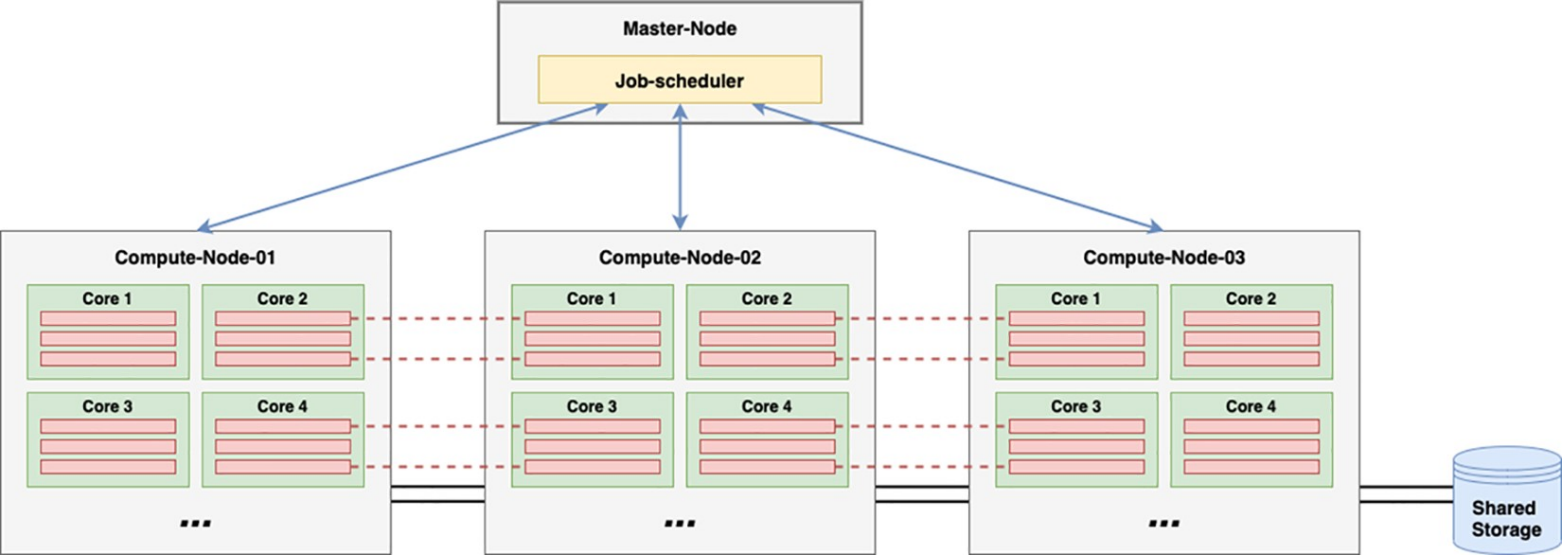
Parallelism in a typical HPC cluster. The job scheduler runs on the master node and controls the highest level of parallelism in running jobs on each node (blue arrows). For example, in a variant calling pipeline, each node may perform the calling for an individual chromosome and could use multiple threads per core—3 threads per core are depicted, for a total of 12 per chromosomal job. In MPI jobs, threads on one node can communicate with threads on other nodes through inter-process communication (depicted in red dashed lines). HPC, high-performance computing; MPI, Message Passing Interface.

In an HPC cluster, the highest level—that is the coarsest granularity of parallelism—occurs at the compute node level, and the lowest level—that is the finest granularity—occurs at the thread level, on each CPU core (Fig 2). In Message Passing Interface (MPI) jobs, processes can communicate with each other across the network through message passing that requires low latency and high-bandwidth interconnects between the compute nodes.

In the Genome Analysis Toolkit (GATK), widely used in genomics and transcriptomics analyses, earlier versions allow for the control of parallelism using the *-nt* and *-nct* parameters that allow the user to specify the number of threads and number of threads per core, respectively. In GATK4, some tools are Spark enabled and can make use of high-throughput distributed multi-threading of the Spark platform—in this case, parallelism is controlled at the thread level and is set using the *-XX*:*ParallelGCThreads* java parameter [8]. These parameters should be set according to the resources allocated for the job and will be dependent on the architecture of the HPC system in use.

### Tip 10: Identify potential bottlenecks in your job

The job scheduler will produce a job report that details the execution time and resource utilisation. Ideally, the report should show high % CPU utilisation—the time during which the CPU executed user commands and a low % system usage—the time during which the system had spent kernel mode executing system processes, e.g., forking, spawning processes, and input/output (I/O) (incidentally, this system time overhead is the reason care should be taken while submitting large numbers of very small jobs. HPC batch schedulers usually allow such types of jobs to be submitted in an aggregated way, i.e., as an array job so that they can optimally manage the system processes involved and scheduling).

The wall time (or real time) of a job is a basic metric that is reported by the HPC job scheduler and can be observed. This is simply the time interval between the job’s start and finish—if your job is taking a long time to run on HPC, it is possible that something might be awry, and it is worth investigating further. The Linux *time* command can be prepended to individual programmes or commands in a batch script and used to monitor the time taken (real time, user time, and system time) for that task to execute.

The types of resource bottlenecks that are likely to occur are the following:

- **CPU bound**: Execution is highly dependent on the speed of the CPU. Rewriting of the algorithm is usually necessary.
- **Disk bound**: Execution time is dependent on disk I/O, e.g., seek times. Your job may be terminated automatically to prevent disk thrashing from occurring. If possible, use a faster storage media, i.e., solid-state drive (SSD). Also, caching data into memory usually helps to reduce repeated disk access.
- **Memory bound**: Execution time is highly dependent on the speed of memory access and is limited by the amount of available memory. Scaling up may help with increasing the available memory per process, but faster access requires faster memory.
- **Network bound**: The network bandwidth is the limiting factor. Where possible, run processes on the same host so as to obviate network traffic.

Inspecting the job’s log files produced by the scheduler is a useful starting point in identifying such problems, although this will report resource usage for the whole workflow script. Performance profiling for individual commands or applications in a workflow can be achieved using the very versatile Linux *perf* command, which is prefixed before the command much in the same way as with the *time* command [9]. The *stat* option of the perf command (i.e., perf *stat*…) is useful, as is the ability to capture system events into a log file with the *record* option—this can be viewed later using the *report* option. However, the monitoring of some system events will require elevated privileges. Where a workflow language is used, the execution engine will typically have an option for producing a report on resource usage for each step of the pipeline—for example, Nextflow offers both resource reporting, producing a detailed HTML document (using the *-with-report* option) and tracing of the individual workflow steps (using the *-with-trace* option), which can provide invaluable information on bottlenecks, as well as rate-limiting steps. Items reported in the Nextflow execution report are CPU usage, memory usage, job duration, and I/O reads/writes.

### Tip 11: Submit your job(s) to the correct queue(s)

On many HPC systems, jobs are submitted singly, or in a batch, to the job scheduler which then adds them to a queue for execution—the type of queue should be chosen by the user, and a default queue is used if one is not specified. Some other system will not let you pick a queue and will pick the “right queue201Dˮ for your job depending its parameters. In the latter case, it is important to understand in which queue the scheduler will send your job to and which nodes, especially how many nodes, are part of that queue. Tweaking your job parameters properly could have a significant impact on the time it spends waiting in queue before it starts running—consult your HPC documentation!

Different types of queue (or indeed partition) are defined in the job scheduler’s configuration by the sysadmin. Separate queues are defined for similar job types, for specific purposes, for example, to define the priority of job execution, to offer different levels of CPU resources and physical random-access memory (RAM), to set a maximum number of jobs per user per queue, to predetermine the maximum duration of job execution, and to allow jobs access to specialised compute node resources, such as GPUs.

It is, therefore, common to see job queues with names such as short, medium, and long—which refer to the maximum wall time (total execution time) or queues named for the specific resources they offer, such as *bigmem*—for jobs requiring large amounts of memory and GPU. Consequently, the use of an incorrect queue—i.e., submitting a job that tries to consume resources in excess of that which the queue provides or a job that exceeds the maximum wall time of the queue—will result in it being automatically terminated by the job scheduler.

Workflow languages, such as Nextflow, often allow the explicit specification of a queue for a particular pipeline step (i.e., process/code blocks), as well as the maximum parallelism for the step. This is useful because the optimal queues can be specified for the task at hand. For example, in the typical case of a workflow that processes a large batch of samples which are generally processed quickly, you may want to submit the main master pipeline script (i.e., parent) job to the long queue. Then, in the workflow script, specify the short (or medium queue) for the step that performs the processing of the sample, the slave (i.e., child) job. This prevents the master job from being prematurely terminated before all of the samples are completed due to exceeding the queues wall time.

### Tip 12: Consider a package manager

A software package management system automates the installation, configuration, upgrading, and removal of software on an OS. For example, Anaconda is a ready to use distribution of data science packages together with a package manager called Conda for any programming language (most commonly Python and R), which is frequently used with Linux and often installed on HPC clusters [10,11]. An initial installation is supplied with Python, Conda, and over 150 scientific packages including their library dependencies. The use of a package manager obviates the need for the user to explicitly manage packages and their configuration, which can often be complex—automatic installation and updating of packages takes care of downloading, updating, and configuring dependencies that are configured in separate Conda environments.

Another important advantage of this approach is that it allows the developer to isolate and manage version dependencies for different projects. For example, when combined with a workflow language, such as Nextflow, the reproducibility of pipelines can be assured by pinning down specific pipeline steps to use specific versions of tools as defined by designated Conda environments. In such a case, the packages defined in a Conda environment can be exported into a YAML file, and this referenced in the workflow step that requires the specific environment. The required packages can then be loaded, or if necessary, downloaded on the fly by Nextflow and cached for subsequent use, thereby allowing workflow pipelines to be shared collaboratively with the confidence that correct packages will be used for execution.

It is important to note that, although use of Anaconda is widespread in HPC, not all sites favour its use. This can be because of the potential for interaction with the existing software environment—this would be dependent on the configuration being used. Additionally, although some Conda packages are optimised for HPC architecture, e.g., Intel SciPy and Numpy packages, others may not be. This is especially true for public repository packages making use of their own MPI libraries. It is therefore necessary to be aware of potential issues. When Anaconda is installed and properly configured for use on an HPC system, it is usually accessed via a module load command. For these reasons, if Anaconda is not made available in this way, it is advised that you first consult your HPC documentation or contact your HPC support team.

### Tip 13: Consider containerisation

Containerisation is a means of virtualising OS environments such that isolated user spaces—containers—can be used to run applications on the same, shared OS. A container is a self-contained, portable environment that contains all the necessary packages and dependencies to run applications within it using an execution engine—the 2 most popular being Docker and Singularity [12].

The portability, encapsulation, and facilitation of reproducibility that containers offer has resulted in the emerging trend to utilise containers on HPC systems. In practice, there are, however, important factors that govern the choice of containerisation engine used. Docker requires a “container daemon” to run the container environment. When a batch script is run by a batch scheduler’s resource manager, the request is made to the container daemon that runs the container as the root user “on behalf of the user.” There are a number of consequences of this, but most importantly, the container then has elevated privileges, and if the batch script stops running, there can be a detachment from the running container. These are not appropriate scenarios for a multi-tenanted HPC system!

Singularity has been developed in order to surmount these issues by not requiring a root-owned container daemon and by restricting the user’s privileges such that the user within the container is the same as the user outside of the container (who submitted and owns the container). Additionally, Singularity has a number of other important characteristics that support HPC. It uses single file–based images, making it friendly for distributed file systems (See Quick Tip 5), supports multiple architectures, and does not require system or architectural changes. Furthermore, it integrates with resource managers, InfiniBand, file systems, and MPI implementations.

Singularity can be used to run the following:

- **applications built for a different Linux distribution** from the host OS;
- **workflows and pipelines in a specific environment** with particular tools and libraries installed within the container (see Quick Tip 14);
- **Singularity or Docker images** without installation—these can be pulled from the Singularity Hub or Docker Hub;
- **open MPI jobs without the need to maintain host lists**—Singularity resource manager integration takes care of spawning as many containers (as MPI instances) as is required to fill the allocation. In this case, *mpirun* is executed outside the container, supplying the container as a parameter; and
- **jobs involving large numbers of small files** can be contained in a single Singularity image—the result is that many accesses to the files within the container maps to only a single access to the HPC file system. This offers improved performance as HPC file systems are, more often than not, distributed and not ideally suited for repeated accesses to very large numbers of small files (See Quick Tip 5).

Typically, Singularity will be made available through the use of a *module load* command. For more information, consult your HPC documentation on the use of Singularity.

### Tip 14: Use a workflow language for your analysis pipeline

Although the HPC batch cluster systems commonly used in research institutes and universities can be used interactively—i.e., through an interactive session—most of the time you will implement scripts to perform certain tasks on some input data, such as when developing an analysis pipeline. A variety of different languages can be used for this, e.g., Python, Perl, or a shell scripting language, such as BASH.

The trouble with this approach, however, i.e., writing collections of scripts de novo to do this, is that such scripts will invariably be platform specific, will need to handle parallelism (particularly managing child jobs and their dependencies), error conditions and, in order to be reproducible, will have to be standardised in some way. Without effectively addressing these issues, which together are often nontrivial, the result is often a script that lacks interoperability, is difficult to reproduce, and will therefore pose challenges in sharing, particularly with external collaborators. A solution for this is to use a workflow language such as Workflow Definition Language (WDL) [13], Common Workflow Language (CWL) [14], Nextflow [15], or Snakemake, an extension to the Python language [16]. This will allow for platform agnosticism, standardisation, and reproducibility, particularly when used with a package manager. See the Ten Simple Rules for Reproducible Computational Research article for some general best practice principles that can be applied when developing and running analysis workflows and computational pipelines [17].

### Tip 15: Use a Jupyter Notebook via an interactive session

The use of Jupyter Notebooks—which facilitates the in-line display of code and its output in a browser (with the code executed within a separate kernel)—has become increasingly popular with scientific researchers [18]. It allows for interactivity and the code to be placed in-line into a narrative context. Although the availability on Jupyter Notebooks on HPC clusters is not yet ubiquitous, its use in this environment is increasing, particularly because it allows for the access to high-performance compute resources and more powerful GPUs than would be commonly found elsewhere. This practice can also help in “bringing the compute to the data,” that is, in performing additional analysis on data that is already present on the HPC cluster.

In the HPC setting, if available, Jupyter Notebooks is usually run via an interactive session (but not the login node, see Quick Tip 3), typically using a Conda environment (See Quick Tip 12). Remote use of Jupyter Notebooks may also be possible, but this will typically require virtual private network (VPN) access or will use X11 forwarding. However, quite often, HPC compute nodes may not have internet access, and so downloading software packages directly could pose a difficulty. Access to GPU resources will often require extra batch scheduler options. Consult your HPC documentation regarding the use of Jupyter Notebooks—there may even be a template script available to get started.
